# Chronic *Cyclospora* infection in a heart transplant patient with intestinal malabsorption: a case report

**DOI:** 10.1128/asmcr.00062-25

**Published:** 2025-08-08

**Authors:** Katie Ambrose, Robert Hamilton-Seth, Mollie Jackson, Rachel Sigler, Daffolyn Rachael Fels Elliott

**Affiliations:** 1 University of Oklahoma School of Community Medicine, Tulsa, Oklahoma, USA; 2 Department of Pathology and Laboratory Medicine, University of Kansas Medical Center, Kansas City, Kansas, USA; 3 Department of Gastroenterology, Hepatology and Motility, University of Kansas Medical Center, Kansas City, Kansas, USA; 4 Department of Infectious Diseases, University of Kansas Medical Center 21638, Kansas City, Kansas, USA; Rush University Medical Center, Chicago, Illinois, USA

**Keywords:** *Cyclospora*, cyclosporiasis, diarrhea, immunocompromised, transplant

## Abstract

**Background:**

Cyclosporiasis is a rare cause of chronic diarrhea and malabsorption in immunosuppressed patients.

**Case Summary:**

We report an unusual case of chronic *Cyclospora* infection in a heart transplant patient with a five-month history of watery diarrhea and weight loss. Initial stool testing failed to discover a causative pathogen. Duodenal biopsy showed histologic findings of intraepithelial lymphocytosis and partial villous blunting, raising the possibility of celiac disease. A repeat duodenal biopsy five months later revealed intracellular parasites with gametocyte, meront, and schizont forms, and the diagnosis of *Cyclospora* infection was confirmed by BIOFIRE Gastrointestinal (GI) Pathogen Panel.

**Conclusion:**

Morphologic diagnosis of enteric parasite infections is challenging and requires a high level of clinical suspicion in immunosuppressed patients. In addition to significant exposures and travel history, chronic immunosuppression should prompt extensive microbial testing, including stool ova and parasite, acid-fast testing, and/or multiplex PCR for GI pathogens in post-transplant patients with unexplained diarrhea.

## INTRODUCTION

*Cyclospora* is a spore-forming, obligate intracellular parasite that can infect the human intestine following consumption of contaminated food or water (1). *Cyclospora* can be transmitted from person to person by the fecal–oral route, and outbreaks of food poisoning have been reported in the United States related to fresh produce (2, 3). Cyclosporiasis is relatively uncommon in the United States, and approximately a third of cases are travel-related to endemic areas in the Caribbean, Central and Southeast Asia, the Middle East, and North Africa (1). People infected by *Cyclospora* may be asymptomatic or develop symptoms of watery diarrhea and abdominal pain. Cyclosporiasis is usually a self-limited diarrheal illness in immunocompetent hosts, although symptoms can persist for several days to weeks without treatment. However, immunocompromised individuals have greater difficulty clearing the infection and subsequently may develop chronic diarrhea, malabsorption, and weight loss (4–6).

Diagnosis of cyclosporiasis can be made routinely by examination of an acid-fast stained stool smear, autofluorescence (7), or genomic methods such as the BioFire Gastrointestinal (GI) Pathogen Panel (8). Notably, these diagnostic methods are not included in routine stool testing at many hospital microbiology laboratories and need to be specifically requested by the clinician. Herein, we present a challenging case of chronic cyclosporiasis in a heart transplant patient on immunosuppression who presented multiple times to our institution with diarrhea and weight loss. The diagnosis was made by histopathology following a second duodenal tissue biopsy for intestinal malabsorption and confirmed using the BIOFIRE GI Pathogen Panel.

## CASE PRESENTATION

### Patient information

A 68-year-old man on chronic immunosuppression presented multiple times for evaluation of diarrhea and 30 pound weight loss over a five-month period. He had a history of orthotopic heart transplantation five years ago for non-ischemic dilated cardiomyopathy, type 2 diabetes mellitus, hypertension, hyperlipidemia, hypothyroidism, and gastroesophageal reflux disease. His maintenance immunosuppression regimen was sirolimus and tacrolimus. Additional medications included levothyroxine, rosuvastatin, tamsulosin, and gabapentin.

His symptoms began during late summer with the sudden onset of watery diarrhea. He reported 5–10 bowel movements per day that were non-bloody, accompanied by intermittent abdominal pain, fatigue, and transient myalgias. He denied any fever, chills, nausea, vomiting, or cough. He did not have any sick contacts or foreign travel. He drank filtered water from an underground well at his lake house during the summer and harvested fresh produce from his vegetable garden.

He initially contacted his outpatient heart transplant team after one week of persistent diarrhea. He tried loperamide for two days without improvement, which was discontinued. He received a 2L bolus of normal saline due to dehydration with low blood pressure (94/66) and elevated creatinine (1.76 mg/dL). One week later, he was admitted to the hospital for further management due to ongoing diarrhea, dehydration, and 12 pound weight loss.

### Clinical findings

Upon admission to hospital, he was afebrile and vital signs were within normal limits. Physical examination revealed a soft, non-tender abdomen with normal bowel sounds. Skin exam showed no rash. Review of systems was unremarkable.

### Diagnostic assessment

Initial stool testing was negative by polymerase chain reaction (PCR) for *Clostridioides difficile*, norovirus, rotavirus, *Cryptosporidium,* and *Giardia* antigens. Stool culture was negative. Of note, ova and parasite (O&P) was requested by the outpatient heart transplant team, but the order was changed per microbiology lab protocol to *Cryptosporidium* and *Giardia* antigen testing due to lack of travel history. Serum PCR testing for cytomegalovirus (CMV) and Epstein-Barr virus (EBV) was negative.

The patient underwent esophagogastroduodenoscopy (EGD) and colonoscopy during his hospital admission. EGD showed duodenal ulcers and erosions. Pathology from the duodenal tissue biopsy reported increased intraepithelial lymphocytes with focal villous blunting. The histologic changes suggested a malabsorptive pattern of injury and raised a broad differential diagnosis, including gluten-sensitive enteropathy (celiac disease), infections, bacterial overgrowth, drug-induced injury, and others. Serologic testing and human leukocyte antigen testing for celiac disease were negative.

His diarrhea failed to resolve three months later, despite lowering immunosuppression, a trial of gluten-free diet, and loperamide. Additional testing revealed elevated fecal calprotectin (108 µg/g; normal range <50 µg/g) and elevated fecal fat (29%; normal range <20%). Abdominal x-ray showed a moderate amount of retained fecal material within the colon. Constipation with overflow diarrhea was considered a possible etiology, followed by an unsuccessful trial of bowel prep cleanse, senna 15 mg nightly (qHS), and increasing fiber intake to 30 g/day with Metamucil.

**FIG 1:**
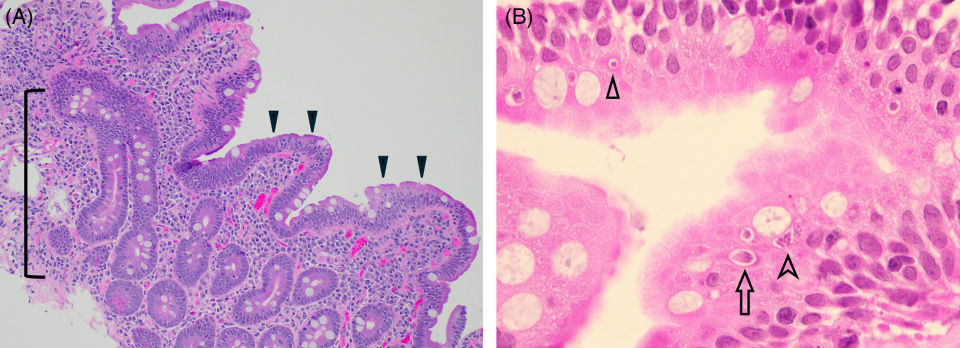
Histopathology of the second duodenal biopsy. (**A**) The biopsy architecture demonstrates partial villous blunting with hyperplasia of the crypts (bracket) and expansion of the lamina propria by lymphoplasmacytic inflammation. There are numerous intraepithelial lymphocytes (arrowheads), ×200 magnification. (**B**) Oil immersion reveals intracellular *Cyclospora cayetanensis* in different stages of the life cycle, including a possible gamete (versus a merozoite in cross-section, arrowhead), a meront (arrow), and a schizont (chevron), ×1000 magnification. A number of forms other than those highlighted are seen in the image as well. Both images: Hematoxylin and eosin (H+E) stain.

Five months later, malabsorptive symptoms of diarrhea and weight loss persisted, so the patient underwent a second endoscopy procedure. EGD showed mucosal changes in the duodenal bulb with flat white plaques. Colonoscopy showed a decreased vascular pattern in the colon. Pathology from the second duodenal tissue biopsy showed increased intraepithelial lymphocytes with crypt apoptosis and focal villous blunting (Fig. 1A). High magnification revealed intracellular round to ovoid structures, 5–10 microns in size, with meront and schizont forms visualized under oil immersion (Fig. 1B). Periodic acid-Schiff–diastase and Grocott’s methenamine silver stains were negative for *Treponema whipplei* and fungal organisms. The histologic findings prompted further microbiologic testing, and the BioFire GI Pathogen Panel multiplex PCR was positive for *Cyclospora cayetanensis*. The overall clinical course is summarized in Table 1 above.

**TABLE 1:** Timeline

Time point	Symptom(s)
T_0_ Time of symptom onset (late summer)	Onset of watery diarrhea
T_0_ + 1 week	Initial outpatient contact with the heart transplant team, endoscopy scheduled
	Laboratory tests: Stool: stool culture, PCR for norovirus GI/GII, rotavirus antigen, *Cryptosporidium* and *Giardia* antigens (O&P order was canceled due to lack of travel history) Serum: PCR for CMV and EBV.
T_0_ + 2 weeks	Admitted to hospital with dehydration and acute kidney injury
	First endoscopy (EGD and colonoscopy) with tissue biopsies
	Duodenal biopsy #1: Histopathology showed increased intraepithelial lymphocytes and focal villous blunting
	Laboratory tests: Stool: stool culture, rotavirus, *Clostridioides difficile*, *Cryptosporidium*, and *Giardia* antigens Serum: PCR for CMV and EBV, thyroid-stimulating hormone, thyroid hormone (T4), serologic testing, and HLA testing for celiac disease
	Intervention: Infectious disease consultation Immunosuppression lowered
T_0_ + 3 months	Imaging: Abdominal x-ray showed a moderate amount of retained fecal material within the colon
	Intervention: Constipation with overflow diarrhea is considered a possible etiology (bowel cleanse, nightly laxative, and increased fiber to 30 g/day)
T_0_ + 5 months	Second endoscopy (EGD and colonoscopy) with tissue biopsies
	Duodenal biopsy #2: Histopathology showed increased intraepithelial lymphocytes and small intracellular ovoid forms that raised the possibility of organisms/spores
	Laboratory tests: Stool: BIOFIRE GI Pathogen Panel positive for *Cyclospora cayetanensis*
	Intervention: Treatment dose TMP-SMX (160 mg/800 mg) twice daily (BID) for 14 days, improvement of symptoms within 48 hours
T_0_ + 7 months	Secondary prophylaxis TMP-SMX (160 mg/800 mg) BID three times weekly for two months, then discontinued with complete resolution of symptoms and no recurrence of diarrhea

### Therapeutic intervention and follow-up

Once the diagnosis of cyclosporiasis was confirmed, the patient was treated with trimethoprim (160 mg)-sulfamethoxazole (800 mg) (TMP-SMX) twice daily (BID) for 14 days, followed by secondary prophylaxis with TMP-SMX (160 mg/800 mg) BID three times weekly for 2 months, and then discontinued. His symptoms improved rapidly within two days of starting therapy. He regained 25 pounds since the completion of therapy and has not experienced any recurrence of diarrhea. His immunosuppression regimen was continued on sirolimus and tacrolimus.

## DISCUSSION

This case demonstrates the diagnostic challenges of an uncommon presentation of *Cyclospora* infection with chronic intestinal malabsorption in an immunocompromised patient. The prolonged duration of infection is likely attributable to his immunosuppressed state after a heart transplant. There are rare case reports of cyclosporiasis in post-transplant patients who suffer relapses of diarrhea and require unusually prolonged treatment (5, 6). Additionally, the ideal duration of secondary prophylaxis in patients on chronic immunosuppression remains undefined (9). It remains uncertain how our patient contracted the infection, but potential exposures included drinking well water and possibly consuming fresh produce from his garden. Interestingly, the onset of diarrhea coincided with his first tomato harvest in late summer. In the United States, *Cyclospora* infection usually occurs after consumption of contaminated fresh produce such as leafy greens and berries, or untreated well water, often during warmer months (1, 2).

Unsporulated *Cyclospora* oocysts are not infective at the time of host excretion. Sporulation occurs 7–15 days following excretion from the host, typically requiring a temperature of 22–30°C (1). Once ingested, *Cyclospora* replicates in the cytoplasm of the enterocyte, with an incubation period of 7 days. The seasonality of cyclosporiasis depends more on human behavioral changes that facilitate transmission than on weather and temperature conditions. Transmission of *Cyclospora* can be reduced through handwashing, avoiding consumption of fresh produce in endemic areas, and keeping workers with GI illness away from fresh produce. Although antifungals and insecticides are not effective against *Cyclospora* oocysts in the environment, magnesium oxide nanoparticles have been found to prevent greater than 90% of sporulation after 24 hours of incubation at 15 mg/mL (10, 11). Currently, the incidence of *Cyclospora* infection is quite low in the United States; however, this could change in the future due to global warming.

One of the challenges of this case was performing the necessary stool testing to make a diagnosis of cyclosporiasis (8). The patient did not meet our laboratory’s required travel screen for O&P testing, with the result that the order was changed to *Giardia* and *Cryptosporidium* antigen testing. Our microbiology laboratory has since expanded the inclusion criteria for O&P testing to include travel or immunosuppression. In addition, modified acid-fast staining, which can be helpful in the identification of oocysts of the coccidian and gregarine parasites (*Cyclospora*, *Cryptosporidium*, and *Cystoisospora*), was originally a separate order at our institution. It has now been merged with the routine O&P testing order. The BIOFIRE GI Pathogen Panel multiplex PCR was recently validated in our laboratory and became available during the timeframe of this patient’s illness, shortly before the second biopsy. The BIOFIRE GI Pathogen Panel can be ordered for inpatients by Infectious Disease, Bone Marrow Transplant, or Gastroenterology clinicians by default at our institution; other services, or providers serving outpatients, are welcome to contact the laboratory to discuss potential cases for testing.

The pathologic diagnosis of cyclosporiasis required two duodenal tissue biopsies in this case and was subsequently confirmed by the BIOFIRE GI Pathogen Panel. Histologic findings of cyclosporiasis in the small intestine can include intraepithelial lymphocytosis, variable villous blunting, and crypt hyperplasia (12). These findings are nonspecific, and additional diagnostic considerations include other infectious etiologies (e.g. adenovirus, CMV, tropical sprue, and bacterial overgrowth), drug-induced injury (e.g. mycophenolate mofetil, non-steroidal anti-inflammatory drugs, and olmesartan), gluten-sensitive enteropathy (celiac disease), sensitivity to nongluten proteins, Crohn’s disease, and immune disorders. A high index of clinical suspicion in the immunosuppressed population and awareness of the histomorphology of parasitic intestinal infections are needed to identify the intracellular organisms.

The most common opportunistic parasites causing severe GI illness in immunocompromised patients are coccidian and gregarine parasites, including *Cyclospora*, *Cystoisospora*, and *Cryptosporidium* species (13). Morphologically, *Cyclospora* oocysts are 8–10 µm in size with several recognizable intracellular life cycle stages, including gametocyte, meront, and schizont forms (2). *Cryptosporidium* has smaller oocysts (3–6 µm) and no merozoite or schizont forms in its lifecycle (8). *Cryptosporidium* is usually apically located with a distinct “bubble-like” morphology, appearing attached to the luminal surface of enterocytes, although the organism is intracellular. *Cystoisospora* has similar lifecycle stages to *Cyclospora*; however, the oocysts (25–30 µm) and merozoite forms of *Cystoisospora* are larger and often seen at the basilar aspect of the enterocyte (8).

Taxonomically, *Cyclospora* belongs to the SAR supergroup, together with other apicomplexan parasites including *Plasmodium*, *Toxoplasma*, and others (7). Historically thought to be a single organism, *C. cayetanensis* has recently been further classified as three separate species, *C. cayetanensis*, *C. henanensis*, and *C. ashfordi*, with differences in geographic distribution and seasonality during which they cause infections (14). The impact of this discovery on our understanding of the clinical course, prevention and treatment of cyclosporiasis is not yet known.

### Conclusion

Cyclosporiasis is a rare cause of refractory diarrhea in the post-transplant population. Immunosuppressed patients can present with clinical, laboratory, and histologic features of intestinal malabsorption that may mimic celiac disease and other common non-infectious GI pathologies. In addition to foreign travel history, chronic immunosuppression should prompt clinicians to order broad-spectrum microbial testing, including stool O&P, acid-fast or autofluorescence microscopy, and/or multiplex PCR for GI pathogens.
